# Deciphering the Role of the Non-Coding Genome in Regulating Gene-Diet Interactions

**DOI:** 10.3390/nu10121831

**Published:** 2018-11-27

**Authors:** Pui-Pik Law, Michelle L. Holland

**Affiliations:** Department of Medical and Molecular Genetics, School of Basic and Medical Biosciences, King’s College London, London SE1 9RT, UK; pui.law@kcl.ac.uk

**Keywords:** non-coding RNA, gene-diet interaction, omics, epigenetics, epitranscriptome, RNA modifications, ribosome, protein translation

## Abstract

Protein encoding genes constitute a small fraction of mammalian genomes. In addition to the protein coding genes, there are other functional units within the genome that are transcribed, but not translated into protein, the so called non-coding RNAs. There are many types of non-coding RNAs that have been identified and shown to have important roles in regulating gene expression either at the transcriptional or post-transcriptional level. A number of recent studies have highlighted that dietary manipulation in mammals can influence the expression or function of a number of classes of non-coding RNAs that contribute to the protein translation machinery. The identification of protein translation as a common target for nutritional regulation underscores the need to investigate how this may mechanistically contribute to phenotypes and diseases that are modified by nutritional intervention. Finally, we describe the state of the art and the application of emerging ‘-omics’ technologies to address the regulation of protein translation in response to diet.

## 1. Introduction

**Figure 1:**
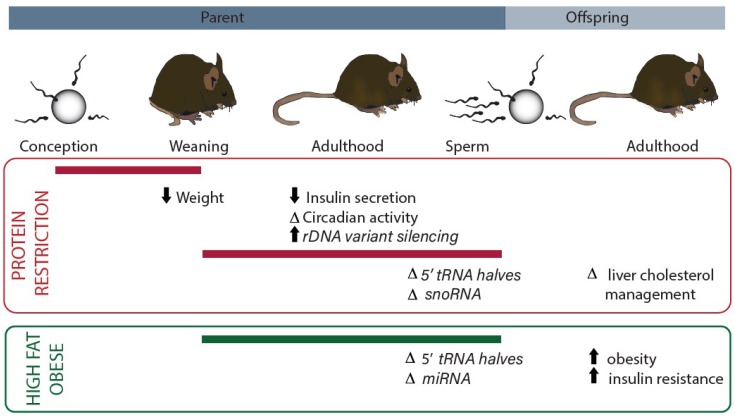
Summary of current evidence for dietary modulation of non-coding RNAs that contribute to the protein translation machinery. Dietary interventions have specific effects depending on the developmental period (indicated with reference to intergenerational life cycle by the solid bar). Studies of protein restriction are indicated in the red outlined panel. Studies of obesity/high fat diet are indicated in the green panel. Measured phenotypes are indicated along with the associated non-coding RNA changes (italics) (rDNA, ribosomal DNA; tRNA, transfer RNA; snoRNA, small nucleolar RNA; miRNA, microRNA).

There is an increasing body of evidence to suggest that nutrition can alter gene expression, with genes that encode components of the cellular protein translation machinery representing a common target (for example: tRNA [1,2,3,4,5], ribosomal protein [6,7], rRNA [8,9,10,11,12,13,14]). The targeting of this pathway is perhaps not surprising given that it is the most energy-consuming cellular process and therefore needs to be tightly coupled to energy availability. Here we will discuss the recent evidence from animal models for nutritional modulation of the non-coding RNA elements that contribute to this machinery and the implications of this for associated phenotypes, namely obesity, insulin resistance and ‘metabolic disease’ (Figure 1). 

We focus on the factors which contribute to the RNA structural components of the ribosome (ribosomal RNA; rRNA) and the small RNAs which direct its site-specific post-transcriptional modification (small nucleolar RNAs; snoRNAs). In addition to ribosomal components, we discuss transfer RNAs (tRNAs), which recruit the amino acids to the site of polypeptide synthesis and their functional cleavage products (tRNA fragments). 

Finally, we address the requirement for further investigation into how nutritional modulation of these components might contribute to disease and outline how the application of a range of recently developed ‘-omics’ technologies can be applied to elucidate how nutrition can interact with underlying genetic variation to modulate gene expression at both transcriptional and post-transcriptional levels.

## 2. Nutritionally Sensitive Non-Coding RNA Species that Contribute to the Regulation of Protein Translation

### 2.1. Ribosomal RNA (rRNA)

rRNAs encoded by the ribosomal DNA (rDNA) are an indispensable structural and catalytic component of the ribosome in which they assist selection of messenger RNA (mRNA) molecules to be translated and catalyse polypeptide bond formation of the amino acids delivered by tRNAs during protein translation [15]. Mammalian genomes encode hundreds of copies of the rDNA operon, arranged in tandem arrays on specific chromosomes (for example: 13, 14, 15, 21, and 22 in human) [16], yet the precise organisation and sequence of these genes is unknown and not included in current genome assemblies. Transcription of rRNA is prolific, accounting for ~35% of the total in the cell [17]. However, only a fraction of the rDNA copies within a cell are actively transcribed due to epigenetic regulation [18,19,20,21,22]. Active copies of rDNA display euchromatic characteristics such as little DNA methylation and the active histone modifications, H3K4 methylation, and H3K9 acetylation. In contrast, silent copies of rDNA are in compact chromatin associated with DNA methylation and repressive histone modifications H3K9 and H4K20 methylation [16]. rDNA copies within a single genome are genetically polymorphic and the number of copies varies between individuals [23,24,25]. Little is known about how genetic variation within the rRNA genes may influence ribosome function. 

Transcription of rDNA is tightly coupled to both the general cellular metabolism and specific environmental challenges [12]. Stress conditions including ageing, cancer and viral infection are associated with reduced rRNA transcription [26]. On the contrary, upregulation of rRNA expression can be achieved by growth factor stimulation [12,26]. Repression of rRNA was also observed in response to different dietary interventions including glucose starvation [8,9,10] and high fat diet [11]. Mutation of genes which encode proteins involved in rRNA biogenesis have been implicated in a number of human diseases [27]. Given that the rate of ribosome production and thus protein synthesis are tightly coupled to cellular growth and proliferation, exquisite control of rRNA expression, the first step of ribosome biogenesis is therefore fundamental for these processes. 

Recently, studies in mice have demonstrated rDNA is a genomic target of early life nutritional insult which may contribute to lifelong phenotypic consequences. Altered DNA methylation and transcription of the rRNA genes in offspring of dams fed a protein restricted diet during particular developmental windows has been shown [13,14]. Diet-induced DNA methylation of rDNA in offspring of mice subjected to protein restriction from conception to weaning was exclusive to a subset of rDNA copies within the genome that could be distinguished by a genetic polymorphism within the rDNA promoter [14]. The extent of DNA methylation at this genetically distinct subset of rDNA correlated negatively with the amount of growth restriction as measured by weaning weight induced by the diet [14]. These mice also displayed reduced spontaneous locomotor activity and reduced glucose-stimulated insulin secretion [14]. Given that birth weight is associated with altered cardiovascular risk factors [28,29,30,31] as well as various type of cancers later in life [32,33,34,35], it will be intriguing to establish if there is a functional link between the extent of diet induced rDNA methylation and disease risk in this model. Importantly, increased rDNA variant-specific DNA methylation was also observed in response to both a high fat and an obesogenic ‘Western’ diet from conception to weaning in the same study [14]. The increased rDNA methylation in this model was independently validated in a separate study, which further identified that this response was specific to a discreet period of protein restriction in early life as continual exposure and exclusively post-weaning exposure did not produce a similar increase in rDNA methylation [5]. These studies identify an interaction between the rDNA genotype and the early life environment which correlates with a distinct phenotypic outcome [5,14]. These results are interesting when interpreted in the light of previous studies showing similar mouse models resulting in altered metabolic phenotypes and increased cardiometabolic disease in adulthood [36,37,38]. 

### 2.2. Small Nucleolar RNAs (snoRNA)

SnoRNAs are short non-coding RNA molecules of around 60–300 nucleotides in length which are found mainly in the nucleolus. The well-conserved snoRNA have been reported in a broad variety of organisms [39,40]. The central function of snoRNAs is direction of 2′-*O*-ribose methylation and pseudouridylation of specific rRNA nucleotides [39]. These modifications are important for correct folding and structural stabilisation of the rRNA [41] and the interaction between rRNAs and other components of the translational machinery [42] as required for normal ribosome function [43,44]. Outside the nucleoli, snoRNA fragments have been reported to act as precursors for functional miRNA [45,46,47,48] and regulators of alternative splicing [49]. Moreover, upregulation of snoRNAs has also been reported under various stress conditions [39] and to be dysregulated in cancers [39,50,51], supporting the physiological relevance of snoRNA function. 

Although direct evidence connecting nutrition to changes in snoRNA expression are limited, a recent study has shown that mice fed a protein-restricted diet from weaning into adulthood have altered snoRNA composition within their sperm small RNA complement [5]. This, together with the suggestion that specific snoRNA-targeted sites in the rRNA are modified on some, but not all molecules, suggests that changes in snoRNA expression could alter the modification profile of rRNA [52]. Alterations in rRNA modification have been implicated in influencing ribosome function [53]. Intriguingly, in lower model organisms, deletion of the rRNA modifying enzyme, (NOP2/Sun RNA methyltransferase family member 5, *NSUN5*) induces alterations in protein translation and organism longevity that only become apparent after a nutritional insult [54]. In mice, the rRNA modifying enzyme Nucleomethylin (*NML*) was shown to be associated with high fat diet-induced obesity [11]. These studies indicate both the importance and convergence of control of protein translation and nutritional modulation of health. 

### 2.3. Transfer RNA (tRNA)

Transfer RNAs (tRNAs) are the most abundant small non-coding RNA molecules making up 4–10% of all cellular RNA [55]. They are well known for their role in delivering amino acids to the ribosome to decode the genetic information on the mRNA for protein synthesis [56]. Beyond this canonical role, tRNAs have also been found to perform additional functions including regulation of global protein synthesis under amino acid starvation [57]. 

Recent studies using high-throughput sequencing have identified small non-coding RNA fragments that are derived from tRNAs [58,59,60]. These tRNA fragments are generated from site-specific cleavage of precursor or mature tRNAs by specific ribonucleases and are divided into distinct categories and sub-categories based on their origin, length, and mapping positions [57,61,62,63]. One class, tRNA-derived fragments (tRFs) are small RNAs of about 14–30 nt in length that map to the ends or the internal region of precursor or mature tRNA [63,64]. A second class of tRNA fragment are tRNA halves which are generated by specific cleavage in the anticodon loop of a mature tRNA molecule to produce 30–40 nt 5′ and 3′ fragments. These tRNA fragments display cell-type specific expression and have important functions, including the regulation of translation through mechanisms that are distinct to the role of mature tRNAs in amino acid delivery [63,64,65]. It is however noteworthy that these tRNA fragments are quite heterogeneous with the multiple possible cleavage sites on the tRNA molecule and the involvement of different tRNA isoaccetpors and isodecoders. 

Numerous tRNA fragment types are elevated under stress conditions, and alteration in their abundance has been associated with various human diseases including cancer and neurodegenerative diseases [62,64]. A number of recent studies based on rodent models have shown adult nutrition influences the relative abundance of specific tRNA fragments in sperm and that this may influence post-fertilisation gene expression, development, and adult metabolic phenotypes in offspring [2,3,4,5]. In the interest of this review, we will focus on reviewing findings involving 5′ tRNA halves which were shown to be associated with dietary intervention. 

Sharma et al. showed that sperm derived from male mice fed a low protein diet display a 2-3-fold increase in multiple types of 5′ tRNA halves (most notably 5′ halves of tRNA-Gly-CCC/TCC/GCC, Lys-CTT and His-GTG) and use 5′ halves of tRNA-glycine-GCC as an example [2]. Knock-down of 5′ tRNA-glycine-GCC halves in mouse embryonic stem cells correlated with upregulation of about 70 genes that are naturally highly expressed in pre-implantation embryos [2]. Interestingly, injection of the small RNA fraction purified from sperm of low-protein fed males (containing increased 5′ tRNA-glycine-GCC halves) reduced the transcript abundance of these same targets in normal two-cell embryos [2]. Adult offspring of these low-protein fed males had altered expression of the transcript encoding a cholesterol biosynthesis enzyme (squalene epoxidase) in the liver [2]. This suggests a mechanistic role for 5′ tRNA-glycine-GCC halves in regulating transcript abundance in early embryogenesis in a manner which might influence cholesterol metabolism in the adult.

Altered expression profile of a subset of 5′ tRNA halves were also observed in the sperm of mice fed a high-fat diet [3]. These 5′ tRNA halves displayed altered RNA modifications, including an increase in 5-methylcytidine (m^5^C) [3]. This specific tRNA modification is mediated by the enzyme DNA (cytosine-5)-methyltransferase-like protein 2 (*DNMT2*), which has been previously implicated in genetic deletion studies as having a role in paternal transmission of putative ‘epigenetic’ phenotypes in mice [66]. Injection of small RNA fractions from the sperm of high-fat fed males into normal zygotes correlated with downregulation of metabolic pathway genes in eight-cell embryos and blastocysts [3]. Adult offspring derived from this technique had altered regulation of glucose homeostasis associated with downregulation of genes involved in metabolic pathways in pancreatic islets [3]. Another independent study also found that offspring of obese sires have no overt phenotypes unless fed a high saturated fat and sugar ‘Western diet’, which led to male offspring having exacerbated fat deposition, glucose intolerance, hyperinsulinemia and hepatic steatosis [4]. Interestingly, these first generation offspring which were not fed a Western diet and appeared metabolically normal had altered miRNA distribution and increased expression of 5′ tRNA halves (derived from tRNA-Gly-GCC, Glu-CTC, Val-CAC and His-GTG) in their sperm and produced second generation offspring that were also hypersensitive to Western diet induced metabolic disease [4]. 

Taken together, these data indicate that gene regulation in the early embryo can be affected by paternal diet via sperm tRNA fragments, with potential consequences for embryonic development and lifetime phenotypes as a result. The molecular mechanisms for how tRNA fragments mediate these alterations in gene expression are still elusive.

## 3. ‘Omics’ Approaches

Increasingly, there are a diverse array of ‘-omics’ approaches available to profile the molecular landscape of choice. These approaches are characterised by being, to some extent, ‘hypothesis neutral,’ that is, to capture all changes that occur in response to an exposure, rather than looking at a particular predetermined molecular target. 

High-throughput sequencing approaches have been developed to profile nucleic acid sequences (DNA and RNA) in a quantitative manner. With the increased cost-effectiveness of sequencing, whole-genome sequencing now allows for the high resolution mapping of inter-individual genetic variation. However, understanding the functional consequences of sequence variation and how it interacts with environmental factors such as nutrition requires correlating genomic sequence variation with changes in the expression, structure, and function of gene products. Furthermore, these relationships are likely to be both cell type and exposure dependent. 

RNA sequencing is a well-established technique and has greatly assisted with understanding how sequence variants can influence the level of transcripts (either mRNA, lncRNA, or small RNAs), leading to the identification of expression quantitative trait loci (eQTLs) [67]. However, the level of mRNA for a particular gene does not necessarily reflect the amount of corresponding protein [68]. Given that the ribosome and other factors involved in the regulation of protein translation have been identified as common targets for nutritional modulation, here we will discuss recently developed techniques that can be used to profile factors which may influence ribosome structure and function, as well as protein translation. Integration of these techniques with well-established DNA and RNA sequencing approaches will be key to creating a more complete understanding of how nutrition and other environmental factors interact with genetic variation to contribute to phenotypes.

### 3.1. Understanding the Consequences of Altered Expression and Post-Transcriptional Modification of Non-Coding RNA Components of the Protein Translation Machinery

As discussed above, there is evidence that both snoRNA expression and tRNA modification and fragmentation can be altered by diet in mice [2,3,4,5], implying that both rRNA and tRNA post-transcriptional modifications are nutrient sensitive. tRNA and rRNA are the most highly modified class of RNAs with about 17% and 2% of their nucleotides being modified, respectively [53,69]. These modifications can impact translational control and are implicated in human diseases [53,55]. 

Some examples in organisms ranging from yeast to human highlight that post-transcriptional modification of rRNA can impact ribosome function in protein translation and may contribute to regulatory functions. The importance of rRNA modifications is demonstrated by the genetic loss of multiple snoRNAs which direct site-specific rRNA modifications causing altered translation efficiency [70,71,72], impaired stop codon termination, and shifts in the translated reading frame [44]. While other changes in rRNA pseudouridylation influence translation initiation from internal ribosome entry sites (IRES) on a specific subset of mRNAs by altering the affinity of the ribosome for these mRNA structures [42,73,74]. In the case of the N1-specific pseudouridine methyltransferase (*EMG1*) gene mutation that underlies the fatal Bowen-Conradi syndrome [75,76], reduced pseudo uridine methylation is associated with a failure of ribosome small subunit assembly [77,78].

tRNA post-transcriptional modifications have also been shown to influence protein translation. Modification of uridine at the wobble position (nucleoside 34) modulates the decoding preference of tRNAs [79], with loss of this modification leading to a reduced rate of protein translation [80]. A range of tRNA modifications have been genetically linked to developmental and metabolic disease. Mutation of NOP2/Sun RNA methyltransferase family member 2 (*NSUN2*), which methylates cytosine-5 of tRNAs is associated with microcephaly in humans and mice [81], while mutation of Threonylcarbamoyladenosine tRNA methylthiotransferase (*CDKAL1*), which catalyses the 2-methylthio-*N*6-threonylcarbamoyladenosine (ms^2^t^6^A) modification of A37 in tRNA-Lys-UUU is associated with increased risk of type 2 diabetes mellitus in humans and mice [82,83,84]. 

These few examples highlight that RNA modifications to the rRNA and tRNA contribute an additional and possibly regulatory mechanism to translational regulation. However, getting a complete picture of the landscape of these modifications is currently limited by the lack of methodologies available to comprehensively map RNA modifications. Here we will discuss techniques to map some specific modifications conferred through snoRNAs and modifications known to be prevalent in tRNAs. Identifying the sites and prevalence of these modifications is an important step in understanding their functional significance.

Pseudo uridine is a highly abundant modified nucleoside found in both rRNA (where its site-specific modification involves the H/ACA family of snoRNAs) and in tRNA [85]. Pseudouridylation in rRNA contributes to translational fidelity and regulation of the translation of specific transcripts that contain internal ribosome entry sites and can undergo cap-independent translation [73]. Recently, two methods for the profiling of pseudo uridine have been developed, both relying on the stable addition of a chemical adduct to pseudo uridine, which causes premature termination of the reverse transcriptase during subsequent library preparation for high-throughput sequencing. These methods allow for the identification of modified sites, as the termination of the sequencing read is significantly enriched at sites of modification compared to libraries prepared from untreated RNA [85,86].

The other snoRNA-mediated rRNA modification is ribose 2′-*O*-methylation (via box C/D snoRNAs), which has also recently been implicated in modulating the capacity for ribosomes to initiate translation from internal ribosome entry sites rather than the 7-methylguanylate cap (m^7^G-cap) [87]. A comparable technique for non-biased mapping of ribose 2′-*O*-methylation sites has been developed. This technique relies on the resistance of the phosphodiester bond 3′ to the site of modification to random alkaline induced fragmentation. Protection from fragmentation leads to under-representation of sequencing reads that start or end at sites of ribose 2′-*O*-methylation [88].

tRNA modifications have been implicated in regulating the level of fragmentation in response to nutritional insults [3]. One of these modifications is 5-methylcytidine [89]. A robust methodology for the quantitative mapping of this modification in RNA has been developed and is akin to busulfite-based profiling of 5-methylcytosine in DNA [90]. This technique relies on the resistance of 5-methyl cytidine residues to deamination by sodium bisulfite, while non-methylated residues are converted. After reverse transcription and library preparation, methylated residues are read as cytosine, whereas unmethylated residues are read as thymine. This is used to determine the methylation status of sites after mapping back to the genome.

There are more than 150 RNA modifications that have been identified, each with a unique set of biochemical properties [69]. So far, only a limited number of modifications have been successfully profiled using a high-throughput sequencing based, genome-wide assay. While the techniques that have been developed represent a significant technological advancement by permitting the identification of novel sites of modification and the relative quantitation (i.e., the stoichiometry), they cannot determine the absolute level of modification or the prevalence of co-modification on a single RNA molecule. The diversity of RNA modifications may mean that obtaining a complete picture of the RNA modification landscape may remain intractable. However, it will be interesting to see how improvements in the capacity for direct RNA sequencing technologies to distinguish RNA modifications will impact this field in the future [91]. 

### 3.2. Understanding the Regulation of Protein Translation by Nutrition

We and others have previously shown that dietary nutrition can influence the expression of distinct genetic variants of the core protein translation machinery [2,5,14]. However, how these effects impact the activity of the translational machinery is unknown. To understand the functional consequences of these diet-induced effects, it is necessary to profile the translatome. The translatome refers to the entirety of mRNA in a cell that is being translated at a given moment and is inferred through an enrichment of specific mRNAs with ribosomes. Two unbiased, high throughput sequencing-based approaches have been developed to date. Both rely on the chemical cross-linking of ribosomes to the mRNA species that they are actively translating. 

Polysome profiling involves size-fractionating the cellular lysate and collecting fractions containing the small and large ribosome subunits, monosomes, or polysomes. Highly translated mRNAs are engaged with multiple ribosomes and will be contained in the heavier polysome fraction. Poorly translated mRNA will be found in the other fractions. The RNA is then extracted from each fraction and used to prepare high-throughput sequencing libraries. The relative enrichment of specific mRNA species in each fraction compared to the non-fractionated input material is then determined [92]. 

An alternative approach that provides higher resolution is ribosome profiling [93]. After cross-linking, the resulting mRNA-ribosome complexes are digested with nucleases, such that only the regions of the mRNA that were bound to a ribosome are protected from degradation. These mRNA fragments are then purified and subjected to deep-sequencing. The density of ribosome footprints is calculated for each mRNA transcript and normalised to transcript length and abundance in the original sample. 

While translatome profiling technologies provide a snapshot, it is necessary to gain an understanding of how both genetic variation and nutritional interventions impact the stable proteome and metabolic status. Quantitative mass-spectrometry based approaches exist to profile proteins or metabolites. The sensitivity of these techniques is ever-improving, permitting detection of an increasing proportion of the abundant components of any biological sample. The integration of these techniques in parallel to the application of the translatome profiling technologies described above is required to understand the link of changes in translational regulation to cellular and, ultimately, organismal phenotypes.

## 4. Summary and Conclusions

It is well established that both genes and environment contribute to health and disease. Here we highlight that the non-coding RNA molecules involved in protein translation present a common target of nutritional modulation. The study of such effects has so far been limited to animal models. This is because even when genetic background is controlled for (e.g., by using inbred mouse strains), the extent of genetic variation in some of these components (e.g., the genes encoding rRNA) is yet to be determined comprehensively. This is due to the genes encoding these elements of the protein translation machinery being present in very high numbers within a single genome. This greatly reduces the ability to produce accurate assemblies of these genes using the relatively short-read sequencing technologies commonly applied to study genetic variation. As such, the extent of within and inter-individual genetic variation within these non-coding RNA is unknown. Even less is known how both genetic variation and nutritional modulation of these components impact the regulation of protein translation. 

Elucidating nutritional regulation of the protein translation machinery will need to be driven by animal models in the shorter term due to the limitations of what is known regarding genetic variation within the key components. However, while long-read sequencing technologies hold the promise to resolve our capacity to map and then explore genetic diversity at these elements, a reverse function-over-form approach can be undertaken simultaneously. Integration of the existing ‘-omics’ approaches to study the translatome and proteome will be useful in establishing just how nutritionally responsive post-transcriptional gene regulation is and what role it might have in directing phenotypes, particularly those associated with altered metabolic function, as indicated from studies to date. While most studies of gene-environment interactions (including the impact of nutrition) to date have focussed on classic ‘epigenetic’ markers associated with altered gene transcription (e.g., DNA methylation), it is highly likely that genetic variation within and nutritional regulation of the protein translational machinery will impact substantially on how an individual will respond to nutritional challenges phenotypically. Now that technology exists to explore these processes, we are posed with an exciting challenge that promises to increase our understanding of how nutrition modifies developmental outcomes and adult disease risk.
